# Head trauma analysis of laboratory reconstructed headers using 1966 Slazenger Challenge and 2018 Telstar 18 soccer balls

**DOI:** 10.1038/s41598-023-45489-2

**Published:** 2023-10-30

**Authors:** Jasmine Ferdousi, Andrew Post, Clara Karton, Klara Doelle, Michael D. Gilchrist, T. Blaine Hoshizaki

**Affiliations:** 1 Health Sciences, University of Ottawa, Ottawa, K1N 6N5 Canada; 2 School of Mechanical and Materials Engineering, University College Dublin, Belfield, Dublin 4, Ireland

**Keywords:** Neuroscience, Diseases, Neurology, Engineering

## Abstract

Retired soccer players are presenting with early onset neurodegenerative diseases, potentially from heading the ball. It has been proposed that the older composition of soccer balls places higher strains on brain tissues. The purpose of this research was to compare the dynamic head response and brain tissue strain of laboratory reconstructed headers using replicas of the 1966 Slazenger Challenge and 2018 Telstar 18 World Cup soccer balls. Head-to-ball impacts were physically conducted in the laboratory by impacting a Hybrid III head form at three locations and four velocities using dry and wet soccer ball conditions, and computational simulation was used to measure the resulting brain tissue strain. This research showed that few significant differences were found in head dynamic response and maximum principal strain between the dry 1966 and 2018 balls during reconstructed soccer headers. Headers using the wet 1966 soccer ball resulted in higher head form responses at low-velocity headers and lower head responses as velocities increased. This study demonstrates that under dry conditions, soccer ball construction does not have a significant effect on head and brain response during headers reconstructed in the laboratory. Although ball construction didn’t show a notable effect, this study revealed that heading the ball, comparable to goalkeeper kicks and punts at 22 m/s, led to maximum principal strains exceeding the 50% likelihood of injury risk threshold. This has implications for the potential risks associated with repetitive heading in soccer for current athletes.

## Introduction

With approximately 265 million participants worldwide^1^ soccer is a contact sport where injuries result from both legal and accidental contact^2,3^. Head injuries are reported as the fifth most common^4^ injury in the sport. As the popularity of soccer continues to rise, research and medical professionals have expressed concerns regarding brain trauma experienced by participants^5^. Head impact events resulting from player collisions, falls, and contacts with the soccer ball contribute to repeated head trauma. Repeated low and high magnitude impacts have been associated with chemical, physical, and neuropsychological changes to the brain that can lead to poor short- and long-term health outcomes^6,7^. In soccer, researchers have reported cases of Chronic Traumatic Encephalopathy (CTE) in deceased retired professional men’s players who were active between the 1960s to 1980s, with many of these individuals competing in the 1966 World Cup^8–11^. With recent media coverage of high-profile retired professional soccer players diagnosed with CTE and other neurodegenerative diseases, there has been an increase in investigations regarding the long-term influence of repeated head impacts from heading in soccer^5,12–16^. One important and unresolved consideration is the construction of soccer balls and how they may affect strains in the brain during heading. It is unknown if modern soccer ball construction will create similar magnitudes of strain as a ball from 1966 during a simulated header.

Soccer is unique, as it is one of the few sports where players intentionally re-direct a ball with their head, a skill referred to as “heading”^17–19^. Heading is the most frequent and repetitive head impact that occurs during soccer games and practices. There have been several biomechanical studies investigating the implications of heading a soccer ball^20–27^. Harriss and colleagues explored purposeful heading in youth soccer and concluded impact magnitudes depend on the game scenario and head impact location^22^. Headers performed at the top of the head resulted in larger rotational velocities compared to the front of the head^22^. Shewchenko and colleagues explored the effect of different soccer balls during heading by manipulating ball mass, pressure, size, and models^26^. They found ball mass reductions of 35% resulted in decreased head accelerations of 23–35%. This study concluded differences in soccer ball characteristics can influence dynamic acceleration head response^26^. While these acceleration measures were used in previous studies to quantify the magnitude of impact, finite element modelling of the brain used to calculate tissue strain is considered a more representative measure of brain trauma than head acceleration or velocity^20–30^. In addition, the velocities of the impacts in previous research were lower than those typically measured during gameplay, which limits their applicability to the range of headers players experience^20,25–27,31,32^.

Differences in the 1966 and 2018 soccer balls may have an influence on strain in the brain tissues during heading in soccer games. As technology advancements occurred, so did the manufacturing and overall design of the soccer ball. In 1966, the Slazenger Challenge was introduced to professional leagues as the first valve-inflated soccer ball^33^. Advanced polyurethane-coated textiles and heat-activated adhesives are now the standards for modern soccer ball construction^34^. Many claim soccer balls are safer now due to new materials, which result in lighter balls^35,36^. However, there is little to no mass difference between present soccer balls to ones manufactured before 1970^26,33^. Furthermore, it is unknown if ball material and overall construction changes are significant when considering strains experienced by the brain as soccer ball advancement has focused on improving play rather than preventing injury^37,38^. Soccer balls made in the 1960s consisted of hand-sewn rubber bladder and genuine leather, allowing them to absorb water during rainy conditions. Shewchenko and colleagues identified these older model soccer balls increased in weight by up to 47% in wet conditions, which lead to higher dynamic head responses compared to the dry ball^26^. They concluded that dynamic head response can be influenced by ball construction, material, and condition^26^. Considering that players from 1966 are reporting negative brain health outcomes that may be associated with repetitive low magnitude brain strains from the heading of a soccer ball, it is important to identify if the construction of the 1966 and modern soccer balls differ sufficiently to affect the strain of the brain tissues. This has implications for current soccer players as heading in soccer is a repetitive activity that may be contributing to brain strains correlated to negative effects on long-term brain health. The purpose of this research was to examine the differences in brain strains from simulated headers using a 1966 Slazenger Challenge used during the 1966 World Cup and the modern Telstar 18 that was used at the 2018 World Cup.

## Materials and methods

### Procedure

**Figure 1:**
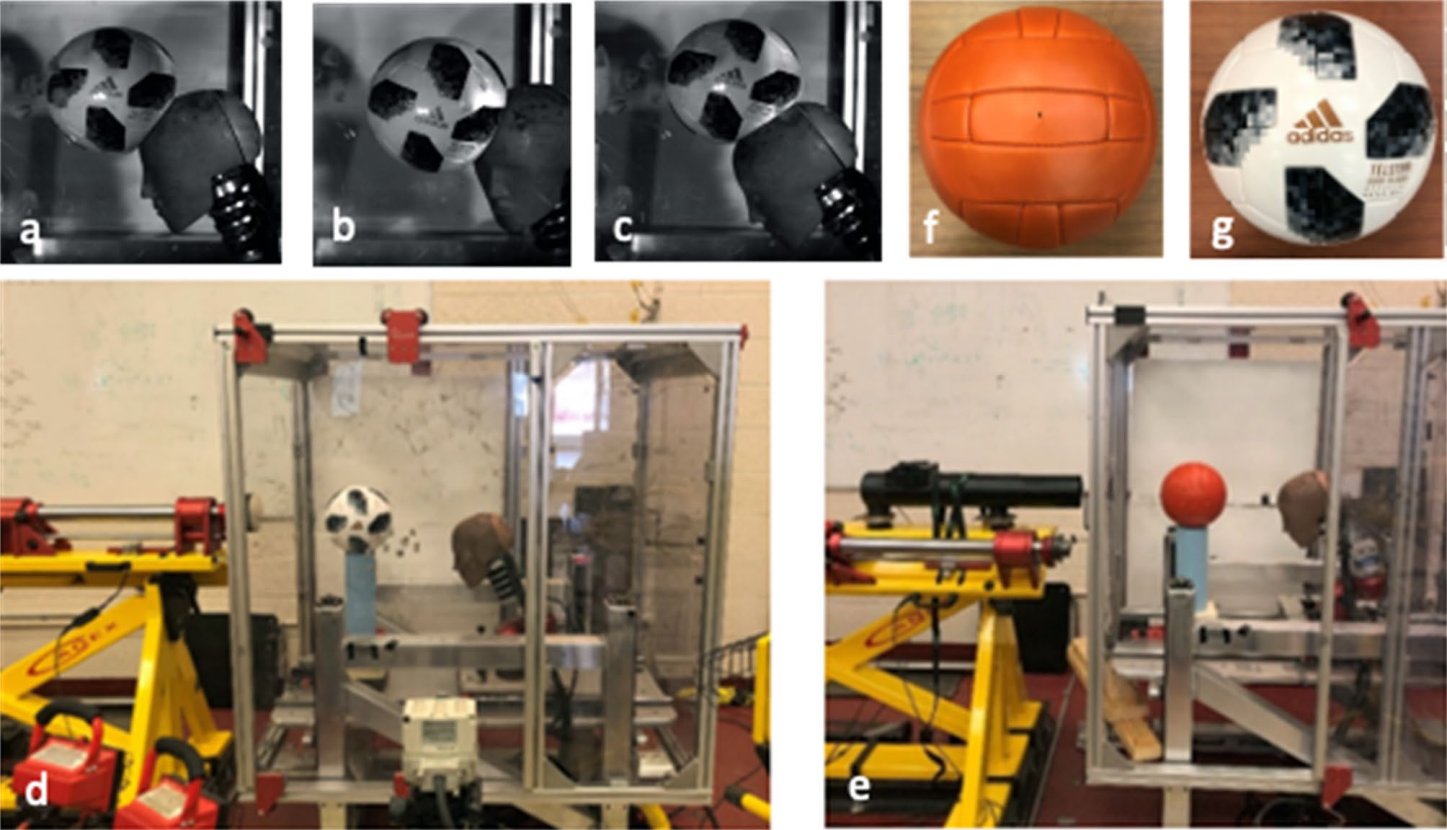
Simulated header impact locations: (**a**) Front; (**b**) Front Boss; and (**c**) Top. The simulated header setup using the linear impactor (**d**) and  projectile launcher (**e**). The 1966 Slazenger Challenge (**f**) and 2018 Telstar 18 (**g**).

Impact parameters for the reconstructed headers were determined from video analysis of 1966 and 2018 soccer games as well as existing soccer literature^20,25,26,39–48^. From this analysis, it was determined that front, front boss, and top were the most common header locations and were subsequently chosen for the head-to-ball impacts (headers) (Fig. 1a–c). Velocities used were based on heading scenarios that were documented in soccer, with the average velocities of 7 m/s, 13 m/s, 17 m/s, and 22 m/s used for the headers (Table 1). Three impact trials per header was physically conducted in the laboratory to obtain linear and angular acceleration data.

**Table 1:** Review of literature to determine ball velocities for simulated headers.

Header scenario	Velocity range in literature	Average velocity	Simulated header velocity
Bounce	6 m/s, 8 m/s^25^ 6.8 ± 0.5 m/s^20^	6.93 m/s	7 m/s
Secondary header	6 m/s, 8 m/s^25^ 6.8 ± 0.5 m/s^20^		
Throw-in	11.7 m/s, 12.4 m/s^40^ 13.4 m/s^41^	12.5 m/s	13 m/s
Pass kick	17.88 m/s^44^ 18.3 m/s^45^ 16 m/s, 21 m/s^46^	16.93 m/s	17 m/s
Corner kick	12 m/s ^42^ 16 m/s^41^		
Punt	20 m/s^43,47^ 25 m/s^43,47^ 22.7 m/s ^48^	21.74 m/s	22 m/s
Goalie kick	25 m/s^43,47^ 16 m/s^42^		

For the laboratory reconstructed headers, the appropriate soccer ball was placed on a cylinder platform between the rod of the linear impactor and the Hybrid III head form **(**Fig. 1d,e). The linear impactor was pressurized to achieve the target velocity, the striker was released to contact the ball at which time the ball would be propelled as a projectile into the head form at the target location. The linear impactor was used to simulate velocities of 7 m/s and 13 m/s (Fig. 1d), whereas the projectile launcher was used for simulations at 17 m/s and 22 m/s because of upper limits on the impactor rod velocity (Fig. 1e). The projectile launcher propelled a weighted softball into the soccer ball and the soccer ball became a projectile that then impacted the head form. A high-speed camera (512PCI Fastcam) was used to confirm the velocity and location of impact for each trial while accelerometers inside the head form measured the resulting linear and angular acceleration curves which were then used as input for the finite element modelling of the brain. For safety, a Plexi-glass enclosure was placed around the sliding table to protect researchers from the soccer ball’s ricochet off the head form (Fig. 1d,e). Each soccer ball was weighed, and pressure was checked at each velocity change. A hand pump with an analog PSI gauge was used to inflate the soccer balls to 10 PSI^49^.

### Wet ball procedure

Tap water was used to fill a large bucket about three-quarters full. Both soccer balls were placed in the water-filled bucket and a concrete slab wrapped in plastic was placed on top of both balls to ensure full submersion. Every 30 min the balls were taken out of the water, wiped down and weighed. This occurred a total of three times at the 30-, 60-, and 90-min time marks. After 90 min the weight of the soccer ball was chosen as the target weight for impacts for the wet condition. The 2018 Telstar 18 ball did not change in weight after 90 min and therefore, did not undergo wet condition impact testing. The 1966 Slazenger Challenge ball weight did change and underwent testing at three velocities (7 m/s, 13 m/s, 17 m/s) at the three head impact locations. Before each impact, the ball was submerged in the water bucket to reach the target weight and pressure was checked to ensure consistency for each trial.

### Equipment

#### Hybrid III head form and neck form

A male 50th percentile Hybrid III head form was used for all head-to-ball impacts (Fig. 1). It has a mass of 4.54 + /– 0.01 kg and was attached to a Hybrid III neck form. The neck form is composed of an internal braided steel cable that passes through the external  rubber neck, which was used to attach it to the linear impactor table.

#### Pneumatic linear impactor

The pneumatic linear impactor used in this study is depicted in Fig. 1d. It consists of a compressed air tank, air cylinder, air release valve, and 13.1 kg impactor rod. The air cylinder propelled the impact rod by discharging the air pressure via a control valve. The air pressure was monitored using a pressure gauge connected to the compressed air tank. An adjustable sliding table was at the end of the linear impactor where the head and neck forms were attached. A hard steel striker was used at the end of the rod.

#### Projectile launcher

The projectile launcher was affixed to the linear impactor frame (Fig. 1e). The impactor rod was rotated downwards to allow attachment of the projectile launcher canon at the top. The projectile launcher used the same compressed air tank, air cylinder, and air release valve.

#### Soccer balls

The 1966 Slazenger Challenge was obtained through World Cup Balls (www.worldcupballs.info), a company that constructs replicas of World Cup soccer balls from 1930 to 1966 (Fig. 1f). This ball was composed of a rubber bladder, genuine leather, and hand stitched, following the soccer ball construction protocols of 1966. The 2018 Telstar 18 was obtained from Spedster Sports (https://spedstersports.com), a company that had the FIFA World Cup Russia 2018 official match ball in stock (Fig. 1g).

#### Data processing and filtering

There were nine Endevco 7264C-2KTZ-2-300 accelerometers (Endevco, San Juan Capistrano CA) arranged in a 3-2-2-2 array within the Hybrid III head form^50^. For the simulated headers, accelerometer data were sampled at 20 kHz with a CFC 1000 low-pass filter. Accelerometer signals were passed through a TDAS Pro Lab system before being processed by TDAS software (DTS, Seal Beach, CA).

#### Finite element modelling

Computational simulation was used to calculate the brain strains for each reconstructed header. The three-dimensional linear and angular acceleration loading curves were used as input to the University College Dublin Brain Trauma Model version 2.0^51^ (UCDBTMv2.0). The head geometry of this model was determined from computed tomography and magnetic resonance imaging of an adult male head and has approximately 184,000 hexahedral elements^52^. The material characteristics of the UCDBTMv2.0 are presented in Table 2^52–55^. The model was validated against pressure responses and brain motion data from impacts to cadavers^51^. Mesh integrity of the UCDBTMv2.0 was conducted to ensure no errors resulted from high aspect ratios^56,57^. In addition, the artificial energy of the simulation associated with hourglassing was monitored and found to be less than 3% of the total internal energy.

**Table 2:** The material properties of the UCDBTMv2.0.

Region	Model	Density (kg/m^3^)	Poisson’s ratio	Parameters
Grey matter^53^	Visco-hyperelastic	1060	~ 0.5	µ = 5715 Pa g_1_ = 0.534, t_1_ = 0.020 s, g_2_ = 0.207, t_2_ = 0.304 s g_inf_ = 0.258
White matter^52^	Viscoelastic	1060	~ 0.5	E = 37,500 Pa g_1_ 0.8, t_1_ = 0.0125 s
Cerebellum^53^	Visco-hyperelastic	1060	~ 0.5	µ = 2611 Pa g_1_ = 0.515, t_1_ = 0.020 s, g_2_ = 0.187, t_2_ = 0.302 s g_inf_ = 0.298
Brain stem^53^	Visco-hyperelastic	1060	~ 0.5	µ = 4768 Pa g_1_ = 0.630, t_1_ = 0.0185 s, g_2_ = 0.175, t_2_ = 0.290 s g_inf_ = 0.290
Pia^52^	Linear elastic	1130	0.45	E = 11.5 MPa
Dura, Falx, and tentorium^55^	Hyperelastic	1130	~ 0.5	µ = 3.602 MPa α = 13.73
Ventricles^52^	Visco-hyperelastic	1040	~ 0.5	C10 = 3653.5 Pa C01 = 4059.44 Pa g_1_ = 0.527, t_1_ = 0.008 s g_2_ = 0.303, t_2_ = 0.145 s
CSF^52^	Linear elastic	1000	~ 0.5	E = 0.15 MPa
Trabecular bone^52^	Linear elastic	1300	0.24	E = 1000 MPa
Cortical bone^52^	Linear elastic	2000	0.22	E = 1500 MPa
Facial bone^52^	Linear elastic	2100	0.22	E = 5540 MPa
Scalp^54^	Hyperelastic (Ogden)	1133	~ 0.5	µ = 1.48 MPa α = 8.1

### Statistical analyses

Statistical analyses were completed using IBM SPSS software. A two-way MANOVA with an α of 0.05 was used to compare linear acceleration, angular acceleration, and maximum principal strain (MPS) values across the simulated headers between the ball conditions.

## Results

**Figure 2:**
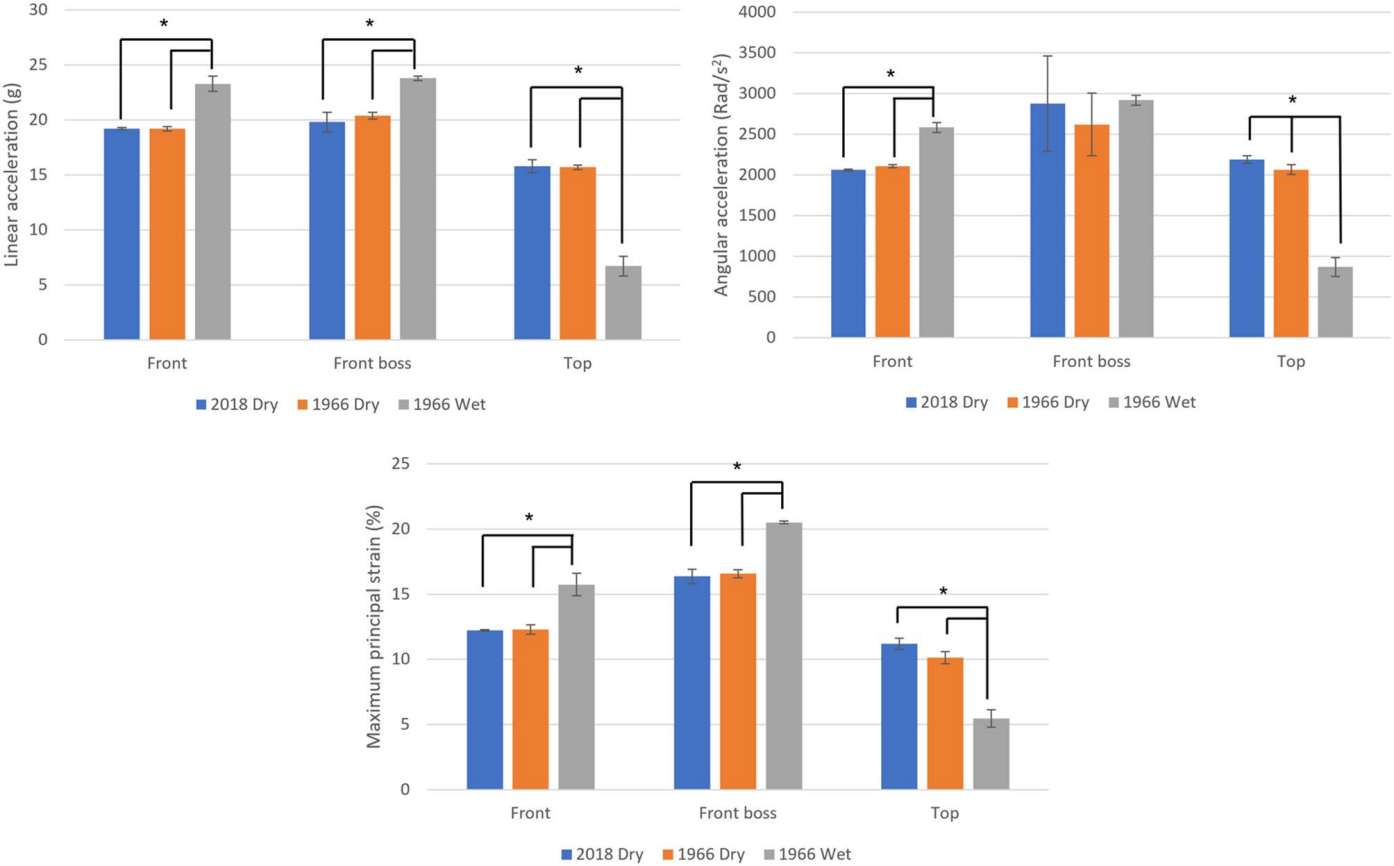
The head from linear and angular acceleration and MPS responses for simulated headers at 7 m/s using 2018 dry, 1966 dry, and 1966 wet soccer balls. Figure shows results of a two-way MANOVA with an α of 0.05. Significance is denoted with *.

Figures 2 through 5 display the average and standard deviation of each dependent variable across different locations and ball conditions per velocity. The 2018 ball did not have a wet condition because it did not uptake water. An example of the resulting head dynamic response time histories is presented for the 2018 dry ball condition to the front location for each impact velocity level, displayed in Fig. 6.

At 7 m/s, the front location head-to-ball impacts with the 2018 and 1966 dry balls had similar responses in linear (*p* = 0.465) and angular (*p* = 0.944) acceleration as well as MPS (*p* = 0.406) (Fig. 2). Impacts with the 1966 wet ball had significantly higher responses than 1966 dry balls for linear acceleration (*p* = 0.027) and MPS (*p* = 0.039). Impacts with the 1966 wet ball also had significantly higher responses than the 2018 and 1966 dry balls for angular acceleration (*p* = 0.038; *p* = 0.026). At the front boss location, impacts with the 2018 and 1966 dry balls have similar responses in linear (*p* = 0.120) and angular (*p* = 0.753) acceleration as well as MPS (*p* = 0.719). Impacts with the 1966 wet ball had significantly higher linear acceleration and MPS responses than the 1966 dry ball (*p* = 0.006; *p* = 0.014). Impacts with the 1966 wet ball also had significantly higher responses in angular acceleration than 2018 dry (*p* = 0.008) and 1966 dry (*p* = 0.004) balls. At the top location, impacts with the 2018 and 1966 dry balls had similar responses in linear (*p* = 0.628) and angular (*p* = 0.935) acceleration as well as MPS (*p* = 0.380). The 1966 wet ball had significantly lower MPS than 1966 dry (*p* = 0.043) ball. There was no significant difference between 2018 dry (*p* = 0.935; *p* = 0.967), 1966 dry (*p* = 0.994; *p* = 0.935), and 1966 wet (*p* = 0.967; *p* = 0.994) balls for angular acceleration at this location (Fig. 2).

**Figure 3:**
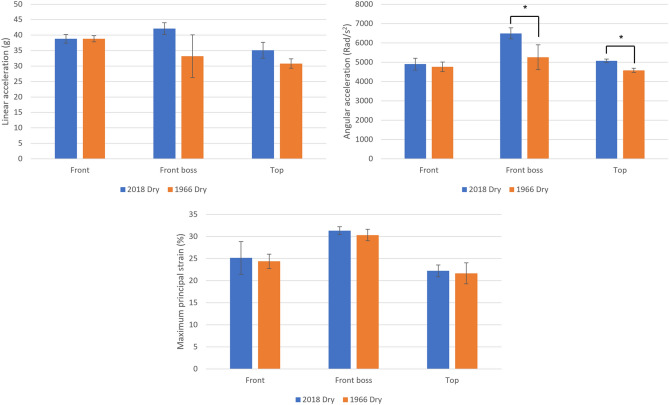
The head from linear and angular acceleration and MPS responses for simulated headers at 13 m/s using 2018 dry, 1966 dry, and 1966 wet soccer balls. Figure shows results of a two-way MANOVA with an α of 0.05. Significance is denoted with *.

At 13 m/s, the front location (Fig. 3) impacts with the wet ball had significantly higher linear (*p* < 0.001) and angular (*p* < 0.001) acceleration as well as MPS (*p* = 0.001; *p* = 0.001) responses than the dry 2018 and 1966 balls. The 2018 dry and 1966 dry balls were not significantly different for linear (*p* = 0.994) or angular (*p* = 0.324) acceleration or MPS (*p* = 0.820). At the front boss location, impacts with the 1966 wet ball had higher linear (*p* < 0.001) acceleration and MPS (*p* = 0.001; *p* = 0.001) responses than the 2018 and 1966 dry balls. The angular acceleration responses were not significantly different between any of the balls (*p* = 0.732; *p* = 0.990; *p* = 0.657). The linear acceleration (*p* = 0.484) and MPS (*p* = 0.791) responses were not significantly different between the 2018 and 1966 dry balls. For the top location, impacts with the wet ball had significantly lower linear (*p* < 0.001) and angular (*p* < 0.001) acceleration as well as MPS (*p* = 0.001; *p* = 0.001) responses than the dry 2018 and 1966 balls. The 2018 dry and 1966 dry balls were not significantly different for linear (*p* = 0.981), angular (*p* = 0.229) acceleration or MPS (*p* = 0.114) (Fig. 3).

**Figure 4:**
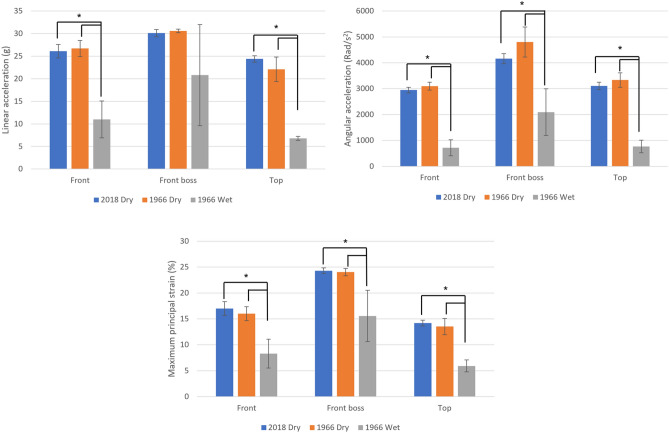
The head from linear and angular acceleration and MPS responses for simulated headers at 17 m/s using 2018 dry, 1966 dry, and 1966 wet soccer balls. Figure shows results of a two-way MANOVA with an α of 0.05. Significance is denoted with *.

At 17 m/s, the front location (Fig. 4) impacts with the wet ball had significantly lower linear (*p* = 0.001) and angular (*p* < 0.001) acceleration as well as MPS (*p* = 0.004; *p* = 0.007) responses than the dry 2018 and 1966 balls. The 2018 dry and 1966 dry balls were not significantly different for linear (*p* = 0.969) or angular (*p* = 0.659) acceleration or MPS (*p* = 0.811). At the front boss location, impacts with the 1966 wet ball had lower angular acceleration responses than 2018 (*p* = 0.024) and 1966 (*p* = 0.027) dry balls. There were no significant differences between the 2018 and 1966 dry balls for linear (*p* = 196), angular (*p* = 0.471) acceleration or MPS (*p* = 0.077). At the top location, impacts with the wet ball had significantly lower linear (*p* < 0.001) and angular (*p* < 0.001) acceleration as well as MPS (*p* = 0.001; *p* = 0.001) responses than the dry 2018 and 1966 balls. The 2018 dry and 1966 dry balls were not significantly different for linear (*p* = 0.267) or angular (*p* = 0.496) acceleration or MPS (*p* = 0.774) (Fig. 4).

**Figure 5:**
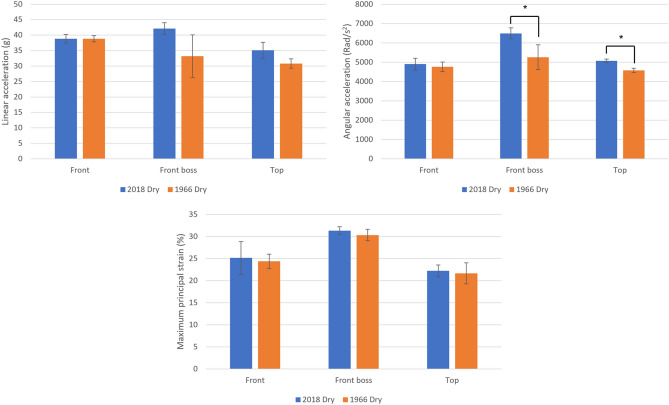
The head from linear and angular acceleration and MPS responses for simulated headers at 22 m/s using 2018 dry and 1966 dry soccer balls. Figure shows results of a two-way MANOVA with an α of 0.05. Significance is denoted with *.

At 22 m/s for the front location (Fig. 5), there was no significant difference found for linear (*p* = 0.975) or angular (*p* = 0.578) acceleration or MPS (*p* = 0.759). At the front boss location, impacts with the 2018 dry ball had significantly higher angular acceleration responses than the 1966 dry ball (*p* = 0.038). There were no significant differences found for linear acceleration (*p* = 0.098) or MPS (*p* = 0.234). At the top location, impacts with the 2018 dry ball had significantly higher angular acceleration responses than the 1966 dry ball (*p* = 0.004). There were no significant differences found for linear acceleration (*p* = 0.068) or MPS (*p* = 0.737) (Fig. 5). Due to equipment constraints, no wet condition impacts were performed at 22 m/s.

**Figure 6:**
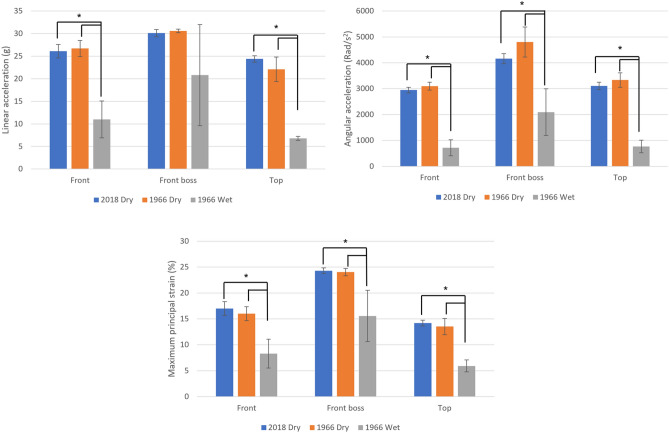
The head from linear and angular acceleration time histories resulting from laboratory reconstructed head-to-ball impacts. The figure displays a front location impact using the 2018 dry ball condition for velocity (**a**) 7 m/s, (**b**) 13 m/s, (**c**) 17 m/s, and (**d**) 22 m/s.

## Discussion

The purpose of this research was to compare laboratory reconstructed headers using a 1966 Slazenger Challenge used during the 1960s and a modern Telstar 18 soccer ball. Specifically, measures of head accelerations and brain tissue strains, as MPS calculated from finite element simulations, were compared. The results show that head-to-ball impacts to the Hybrid III head form using the 1966 Slazenger Challenge and the 2018 Telstar 18 resulted in similar linear and angular accelerations and MPS for all dry ball impact conditions except for angular accelerations at 22 m/s (Figs. 2–5). The reduction in angular acceleration that occurred during impacts at 22 m/s with the 1966 dry ball may be because of the unique exterior stitching of the 1966 ball, which could have affected the impact. However, using either soccer ball, headers representing punts and goalie kicks at velocities of 22 m/s, produced magnitudes of angular acceleration and brain tissue strain response within the reported range of a 50% probability risk of concussion and structural damage to neurons^28,58–62^. In general, the results demonstrate that there were few clinically relevant differences in head impact magnitude for the current international standard soccer ball when compared to the hand-sewn leather soccer ball used in 1966.

A comparison of head form impact responses between the wet 1966 and dry 1966 and 2018 balls showed that the wet ball consistently resulted in higher linear and angular accelerations as well as MPS for front and front boss impacts at 7 m/s and 13 m/s. These results are consistent with previous literature demonstrating an increase in head response when the mass of the ball is increased^26^. The exception to this was found at the top location where the 1966 wet ball measured significantly lower linear and angular acceleration as well as MPS when compared to 2018 dry and 1966 dry balls (Figs. 2, 3). Furthermore, the 1966 wet condition showed significantly lower linear and angular acceleration and MPS measures when compared to 2018 dry and 1966 dry at 17 m/s for all locations (Fig. 4). This was interesting as the weight of the ball for the wet condition increased from 410 g to 595 g after 90 min of water submersion. Studies that have explored older model soccer balls and tested wet conditions have documented an increase in dynamic head response with an increase in soccer ball weight when participants outfitted with instrumented mouthguards headed the ball at velocities of 6 m/s and 8 m/s^26^. Using high-speed video analysis in our study it was observed that with the combination of Hybrid III head form geometry, the wet ball condition, and high velocity, the ball slid off the head form rather than resulting in a direct impact, potentially contributing to the reduced head dynamic response. This may explain the lower responses for wet ball impacts at these velocities and locations. Live human subjects likely adjust for the slippery surface of this condition, engaging in better contact between forehead and ball.

The results indicate the 1966 Slazenger Challenge and the Telstar 18 soccer balls, when dry, produce similar magnitudes of strain from simulated headers. Retired players who played in 1966 are currently presenting with symptoms of neurodegenerative diseases that have been associated with the repeated heading of the ball^5,12,13,16^. In this study, the lowest heading velocity tested of 7 m/s, produced MPS results of 5–10% strain, which increased to 20% strain and beyond with increasing header velocity. Low magnitude strains have been associated with metabolic and structural changes in affected neurons that, if repetitive, may lead to long-term negative brain health outcomes, such as CTE, and other neurodegenerative diseases^42,43^. This study demonstrates that specific header scenarios lending to higher ball velocities, produce strain levels consistent with reported levels of neuronal damage. However, repetitive traumas are experienced from various forms in soccer, including player to player collisions and falls to the ground. It is important to include all sources of brain trauma when considering a player’s overall exposure and cumulative damage manifestating as brain disease and various neurological outcomes over time. The inclusion of headers and non-headers seems an appropriate focus of future work.

This study is not without limitations and should be considered when interpreting the results. The actions of heading in real-life games are slightly different from the head-to-ball impacts reconstructed in the lab. One factor is the head form was not moving toward the ball as players would during a header. Additionally, players often redirect the ball by moving their head from one side to the other; the head form in the lab was stationary until impact. The UCDBTMv2.0 is a finite element model and provides responses based upon its material characteristics and boundary conditions, and while validated, will not produce strains identical to those that may be incurred by soccer players heading the ball under real-world conditions. As a result, the magnitudes of strain should be considered representative of the severity of impact to the head with a ball.

## Data Availability

The datasets used in the current study are available from the corresponding author upon reasonable request.
